# Alternative Methods for Measuring the Susceptibility of White Wines to Pinking Alteration: Derivative Spectroscopy and CIEL*a*b* Colour Analysis

**DOI:** 10.3390/foods10030553

**Published:** 2021-03-07

**Authors:** Fabrizio Minute, Federico Giotto, Luís Filipe-Ribeiro, Fernanda Cosme, Fernando M. Nunes

**Affiliations:** 1 Giottoconsulting srl, 31051 Follina, Italy; fabrizio@giottoconsulting.it (F.M.); federico@giottoconsulting.it (F.G.); 2 CQ-VR—Chemistry Research Center—Vila Real, Food and Wine Chemistry Laboratory, University of Trás-os-Montes and Alto Douro, 5000-801 Vila Real, Portugal; fmota@utad.pt (L.F.-R.); fcosme@utad.pt (F.C.)

**Keywords:** pinking, white wine, derivative spectroscopy, CIEL*a*b* colour space

## Abstract

Pinking is the term used for describing the pink colouration that appears in white wines produced under reducing conditions when oxidised. The ability to predict the susceptibility of white wines for pinking is of utmost importance for wine producers. In this work, we critically compare the two most currently used methods for measuring pinking susceptibility and the use of the first derivative spectra and the CIEL*a*b* colour space method. The amplitude of the first derivative spectra in the 450–550 nm range has a good correlation with the values obtained by subtracting the extrapolate background at 500 nm (R^2^ = 0.927); therefore, first derivative spectroscopy seems to be a more straightforward approach for eliminating the background problem that occurs in this method. The CIEL*a*b* method using the a* value after oxidation seems to be the most appropriate method to measure the pinking susceptibility of white wines, showing a very good correlation with the amplitude of the first derivative spectra. The pink colouration visualisation is linearly related to the b* value of the white wine, showing that no universal cut-off value for predicting the pink visualisation should be used. Second derivative spectra allow the observation of the formation of different chromophores in wines after induced oxidation.

## 1. Introduction

Wine colour is one of the first parameters evaluated by consumers and contributes to the wine’s quality perception and acceptance [1]. In fact, tasting is strongly influenced by wine colour much more markedly than other chemical–sensorial parameters [2]. It is therefore clear that the colour stability of wines in the pre-and post-bottling phases is a key aspect to be taken into account.

The most common colour defect in white wines is browning, a chromatic alteration that gives rise to a brown colour related to the oxidation of flavan-3-ol derivatives [3]. In recent years several authors have focused their attention on the characterisation of the chemical processes underlying browning, identifying several possible solutions to prevent or correct this problem [4,5,6]. A rapid test to assess browning development, known as the “Polyphenol Oxidative Medium test” (POM-test), was also proposed [7].

Much less information is available in the literature regarding another chromatic alteration of white wines which is manifested by the appearance of a pinkish colour. This phenomenon is known as pinking [8]. Especially observed in young wines produced under reducing conditions, it occurs after exposure to oxygen with a more or less marked intensity which seems to be related to the white grape variety and harvesting year. The origin of pinking in white wines is still a matter of debate. It has been described that the appearance of the pink colour in white wines is derived from at least 10 compounds and polymeric material [9]. Several hypotheses have been developed for explaining the appearance of the pinking colouration. One of the first hypotheses is that the pinking phenomenon may occur due to the slow dehydration of leucoanthocyanidins (flavan-3,4-diols) to their corresponding flavenes (flav-3-en-3-ol) under a highly reductive medium and then quick oxidation to their corresponding coloured flavylium cations (cyanidin) upon exposure to oxygen [10,11]. Another hypothesis is attributed to the slow acid catalysis cleavage of interflavan bonds of certain proanthocyanidins present in grape skins to their corresponding carbocation intermediate which, following an oxygen exposure, turn into flavylium cations [8]. Additionally, the pink colour developed in white wines has been attributed to the formation of unknown red coloured compounds after oxidation of the 2-*S*-glutathionilcaftaric acid [12]. The more recent hypothesis is that the pinking of white wines is due to the presence of small amounts of anthocyanins in white grapes that are extracted and after a reversible reaction with the hydrogen sulphite ion resulting from the use of sulphur dioxide at the crushing of grapes renders the flavene-4-sulfonate that is colourless. When the amount of sulphur dioxide in the wines lowers, for example, during storage or upon exposure to oxygen, there is observed an increase in the flavylium form of the anthocyanins in the wine due to the dissociation of flavene-4-sulfonate. When the concentration of the coloured form of anthocyanins reaches a certain concentration, the pink colour can be visually detected in the white wine [13,14].

At present, there are no treatments to fully reverse pinking once occurring. A partial reversion has been shown after exposure of pinked wine to ultraviolet (UV) light, although this treatment is not efficient enough to reach the initial wine colour [15]. Therefore, preventing pinking appearance is at the moment the best approach. The most common treatments for the prevention of pinking are the use of ascorbic acid before bottling or fining wines with polyvinylpolypyrrolidone (PVPP) or potassium caseinate that can adsorb the precursors responsible for the colour alteration [16]. The dosage of fining agents has to be finely tuned taking into account also the impact on the wine flavour and aroma [17]. Therefore, methods for measuring the susceptibility of white wines to pinking have been developed and widely used. A method based on the measurement of the optical density at 500 nm of a wine treated with hydrogen peroxide was proposed by Simpson [8]. As reported by Simpson [8], these conditions also determine an increase in the signal at 420 nm commonly used to evaluate another chromatic phenomenon typical of white wines: browning. Considering that the exposure of wine to more or less marked oxidative conditions generally determines an increase in both optical densities, possible errors in the interpretation of the results may arise. A solution to this problem was also presented by Simpson [8] by extrapolating a baseline and measuring the difference in the optical density at 500 nm and the baseline value at the same wavelength after forced oxidation. Derivative spectroscopy can be a solution to cope with this difficulty. The value of the signal in derivative spectroscopy depends on the shape of the basic spectrum. For sharp peaks, the signal after differentiation increases with that of the derivative order, contrary to flat peaks, for which the signal decreases. The ratio of signals is inversely proportional to the ratio of peak half-width in an order equal to that of the derivative. This allows exposing a small sharp peak overlapped by a flat, even intense band from the total basic spectrum. On the other hand, the CIEL*a*b* colour space is the standard International Organisation of Vine and Wine (OIV) method for the determination of the chromatic characteristics of wine [18]. This method allows the reproduction of the visual sensation of colour perceived by observers through the use of three chromaticity coordinates: lightness (L*), red/green colour component (a*), and blue/yellow colour component (b*). Compared to classical optical density, CIEL*a*b* parameters offer a more precise description of wine colour [19,20].

The purpose of this work is to critically evaluate alternative methods for evaluating the pinking susceptibility of white wines including the use of derivative spectroscopy for the elimination of the brown background in the visible spectra and the CIEL*a*b* colour space for measuring the increase in the red (a*) component induced by forced oxidation.

## 2. Material and Methods

### 2.1. Wine Sample Selection

The wines used for pinking tests were listed by GiottoConsulting srl client companies of different regions (Table 1) on the basis of the susceptibility to pinking highlighted over the years. All the wines used for the tests were vinified in the same year in which the tests were carried out. The vinification protocols are the standard ones developed by the various companies and considered the best possible solutions in terms of quality of the finished product. Considering the measures adopted to protect wines from oxidation, especially during must extraction, the vinification protocols adopted for the wines can be grouped into two macro-groups: “classic” and “reductive” vinification (Table 1). In the “classic” vinification the grapes were not pressed in a hyper-reductive environment and were protected with the addition of potassium metabisulfite and nitrogen in the press, then musts were floated and fermented in stainless steel or concrete tanks. In the “reductive” vinification, wines were vinified in more extreme reductive conditions starting from cold grapes (dry ice used) and using inertised presses. Then musts were clarified by flotation or cold decantation and fermented in stainless steel or concrete tanks. Regardless of the vinification protocol adopted, at all stages of winemaking free sulphur dioxide was carefully monitored to avoid oxidative phenomena. The chemical parameters of wines (Supplementary Material Table S1) were determined by Fourier Transform Infrared spectroscopy using an FT-120 (Foss).

### 2.2. Measurament of the Pinking Susceptibility of White Wines

The pinking susceptibility of white wines was evaluated as described by Simpson [8], by adding 0.25 mL of H_2_O_2_ at 0.3% (*v*/*v*) to 10 mL of white wines. After 24 h of incubation in the dark at room temperature (20 °C), the pinking susceptibility of white wines was quantified as described in Section 2.4.

### 2.3. Accelerated Pinking Test

To overcome the time required to perform the test proposed by Simpson [8] (24 h), a quick test was proposed by Ferrarini (unpublished data) that allows to evaluate the pinking potential in just 30 min. To 10 mL of white wine in a test-tube with a stopper, 0.25 mL of H_2_O_2_ at 0.3% (*v*/*v*) was added. The test tube was tightly closed and incubated in a water bath (Memmert Basic WBN) at 60 °C for 30 min. The samples were filtrated through a 0.45 µm filter before (and after if wine presents protein instability) the test. Visible absorption spectra were obtained using a Cary 60 spectrophotometer (Agilent Technologies, Santa Clara, CA, USA) using a 1 cm cell from 380 to 780 nm with a 1 nm resolution.

### 2.4. Quantification of the Pinking Susceptibility

The two classic methods proposed by Simpson [8] were used for quantification of the pinking susceptibility: (1) by measuring the absorbance at 500 nm before and after the test and multiplying by 1000, and (2) by extrapolating the baseline at 500 nm from the use of an exponential fitted equation to the absorbances at 650, 625, 600, 420, 410 and 400 nm and subtracting this theoretical baseline at 500 nm to the value obtained at 500 nm and multiplying by 1000. According to Simpson [8] if the value obtained is less than 5, then the wine is not susceptible to pinking.

### 2.5. Derivative Spectroscopy

Derivative spectroscopy is based on the use of the derivative spectra of a zero-order spectrum. The derivative spectrum and can be expressed as:

^n^D_λ_ = d^n^A/dλ^n^ = f(λ)

where: n—derivative order, ^n^D_λ_ represents the value of the n-order derivative of an analyte at the analytical wavelength (λ), A—absorbance.

The most important properties of derivative spectrophotometry, similar to those in classic spectrophotometry, are the dependence of the derivative value on concentration and its additivity. By differentiating the expression for the Lambert–Beer law over the wavelength, the following equation is obtained [21,22]:

^n^D_λ_ = d^n^A/dλ^n^ = d^n^ε/dλ^n^.c.l

where ε—molar absorption coefficient (cm/mol/L), c—concentration of an analyte (mol/L), l—thickness of solution layer (cm). The derivative spectrum of the n-component mixture is a sum of derivative spectra of individual components:

^n^D_mix_ = ^n^D_1_ + ^n^D_2_ + … + ^n^D_n_

A useful feature of derivative spectroscopy is the dependence of derivatisation results on the geometrical characteristic of the starting, zero-order spectrum. The shape and the intensity of the resulting derivative spectrum depend on the half-height width of the peaks in the zero-order spectrum. Due to this property, broad zero-order spectra are quenched with the generation of higher orders of derivatives, while narrow peaks undergo amplification. If the zero-order spectrum possesses two bands, A and B, which differ from their half-heights width (LB > LA), after a generation of n-order derivative a ratio of derivatives intensity can be expressed as:

^n^D_A_/^n^D_B_ = (L_B_/L_A_)^n^

Derivative spectrophotometry provides an increase in sensitivity and selectivity of the methods in comparison with the classical derivative spectrophotometry based on the same colour system. An increase in selectivity in the derivative spectrophotometry results from the fact that differentiation permits to obtain a larger amount of information contained in the basic absorption spectrum. Due to this, it is possible to take advantage of the differences in the position of the peaks (different A.max) and in the peak half-width (different L values) [21,22]. For obtaining the first derivative and second derivative spectra the Savitzky–Golay algorithm [23,24] was used with 20 points smoothing and a 3rd order polynomial using Origin 8 software (Origin, OriginLab Corperation, Northampton, MA, USA).

### 2.6. CIELab Colour Measurement

Wine’s chromatic characteristics before and after induced oxidation by the addition of hydrogen peroxide were determined using the CIELab method according to OIV [18]. The chromatic parameters (a*: red/green; b*: yellow/blue; and L*: lightness/darkness) were obtained. Differences in chromatic parameter before and after the test were calculated as:

Δa* = a* before test − a* after test;

Δb* = b* before test − b* after test;

ΔL* = L* before test − L* after test;

### 2.7. Determination of the Colour Threshold for Pinking Perception

Wines of different varieties and ages were used to determine the perception threshold of pink. In a volume of 50 mL of each wine, increasing volumes (10 to 478 µL) of a deep red wine (Rossissimo) were added, and then the visible spectra were acquired as described above.

Sensory analysis was performed by a panel composed of nine experts, oenologists, and laboratory technicians who over the years have faced the problem of pinking in wines of various grape varieties and origins. Judges were asked to number the solution where a pink colouration was observed. Each tube corresponded to a different Δa*, and the perception value of the pink colour was taken as the average between the nine panelists. The consistency between panelists (C-index) was evaluated by consonance analysis [25], performing a principal component analysis on the panel data. Good agreement between panelists was indicated by high C-index values (>1) [26].

## 3. Results

### 3.1. Pinking Susceptibility Calculation Method Comparison

**Figure 1:**
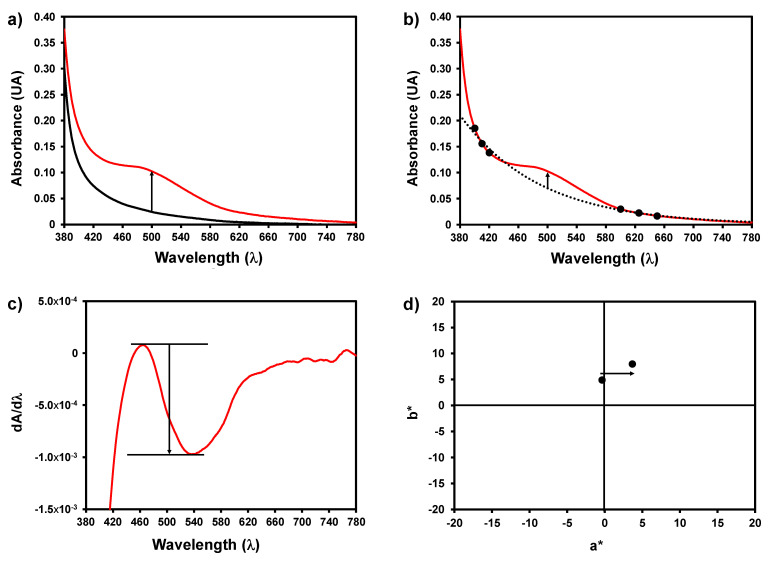
Illustration of the four procedures used for determination of the pinking susceptibility of white wines. (**a**) Difference in absorbance at 500 nm before and after oxidation; (**b**) difference between the absorbance at 500 nm and the baseline value at the same wavelength extrapolated by using an exponential fitting to the values obtained at 650, 625, 600, 420, 410, and 400; (**c**) amplitude of the first derivative spectra in the 450 to 550 nm region; (**d**) CIEL*a*b* chromatic characteristics of the wines before and after oxidation.

**Figure 2:**
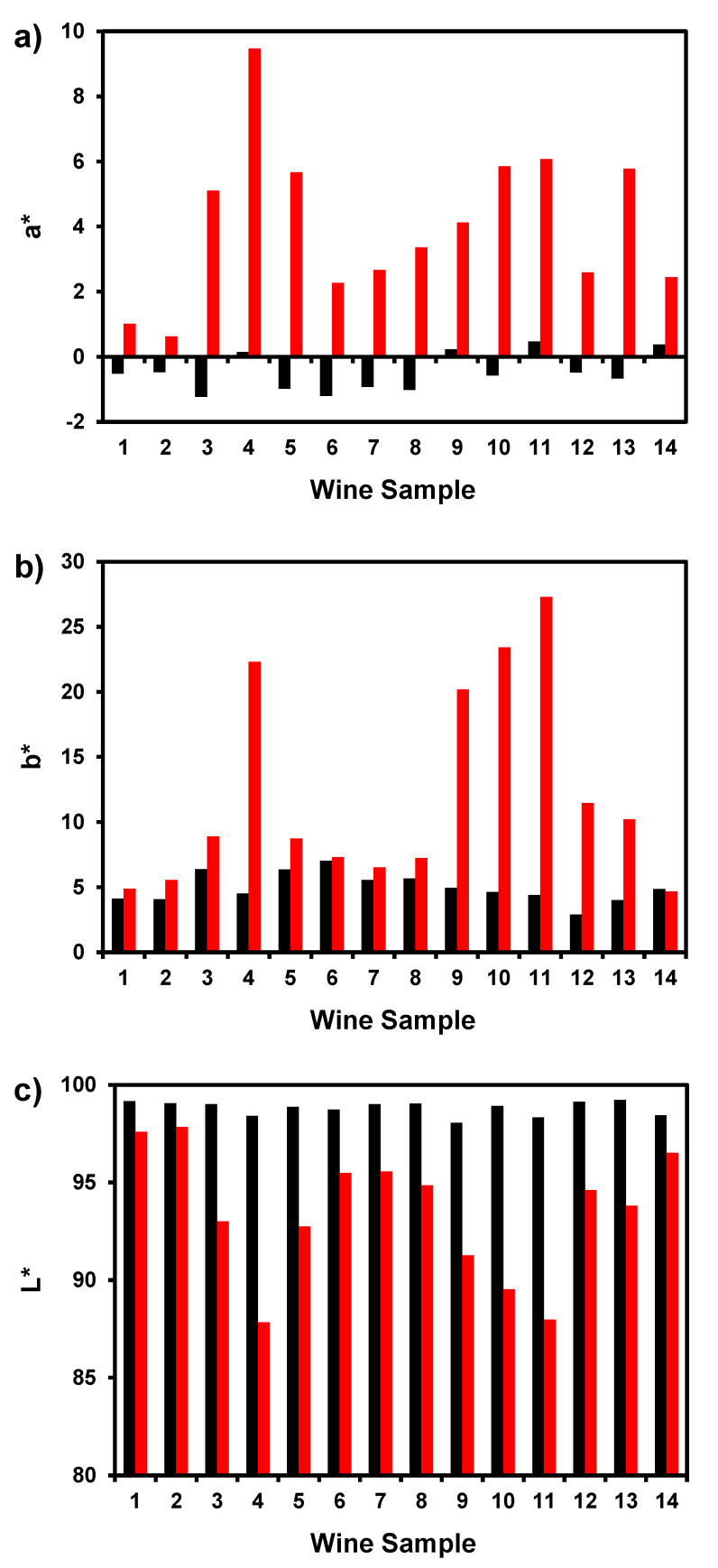
CIEL*a*b* chromatic parameters before (black bars) and after (red bars) the induced oxidation of wines with hydrogen peroxide at room temperature for 24 h. (**a**) Red/green colour component (a*) values; (**b**) Blue/yellow colour component (b*) values; (**c**) Lightness (L*) values.

A set of 14 wines with different pinking susceptibilities, obtained from different white grape varieties and countries, were used in this study. The chemical parameters of the wines are shown in Supporting Information Table S1. The pinking susceptibility of the wines was measured by inducing oxidation of wines after the addition of hydrogen peroxide as described in the material and methods section. The visible spectra were obtained from 380 to 780 nm with a 1 nm resolution. Four methods were used to estimate the increase in the absorbance in the 500 nm zone, using the classic approach described by Simpson [8] either (1) by measuring the increase in absorbance at 500 nm before and after oxidation (Figure 1a) or (2) by extrapolating the brown colour baseline using an exponential fitting to the values obtained at 650, 625, 600, 420, 410 and 400, and obtaining the baseline value at 500 nm (Figure 1b). The results obtained were compared to those obtained using the first derivative spectroscopy of the spectra after pinking phenomena (Figure 1c) and by calculating the chromatic characteristics of the wines before and after oxidation (Figure 1d).

The wines studied showed a high variability of CIEL*a*b* chromatic parameters with b* values ranging from 2.899 to 7.024 (Figure 2b) probably related to the different grape varieties used and winemaking procedures. On the other hand, a* values presented negative values in almost all the wines, showing that the red component was absent in these wines (Figure 2a). The treatment of these wines with hydrogen peroxide at room temperature for 24 h resulted in an increase in both a * and b* parameters and a decrease in the L* parameter. Prosecco was the wine analysed with a lower variation in the a* value while Sauvignon Blanc was the wine with the highest variation of the a* value (Figure 2a–c).

**Figure 3:**
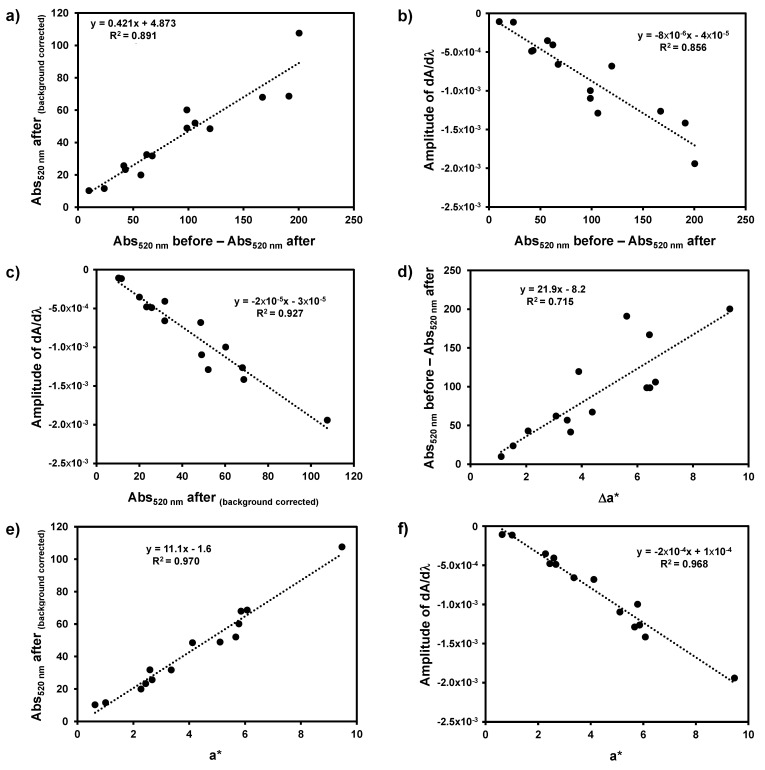
Comparison of the pinking susceptibility values obtained by the four methods evaluated in this work. (**a**) Difference in absorbance at 500 nm before and after oxidation versus the difference between the absorbance at 500 nm and the baseline value at the same wavelength extrapolated by using an exponential fitting to the values obtained at 650, 625, 600, 420, 410, and 400; (**b**) difference in absorbance at 500 nm before and after oxidation versus amplitude of the first derivative spectra in the 450 to 550 nm region; (**c**) difference between the absorbance at 500 nm and the baseline value at the same wavelength extrapolated by using an exponential fitting to the values obtained at 650, 625, 600, 420, 410, and 400 versus amplitude of the first derivative spectra in the 450 to 550 nm region; (**d**) difference in absorbance at 500 nm before and after oxidation versus Δa*; (**e**) difference between the absorbance at 500 nm and the baseline value at the same wavelength extrapolated by using an exponential fitting to the values obtained at 650, 625, 600, 420, 410, and 400 versus a*; (**f**) amplitude of the first derivative spectra in the 450 to 550 nm region versus a*.

In Figure 3a, the correlation is shown between the values obtained for pinking susceptibility by measuring the increase in absorbance at 500 nm before and after oxidation and the values obtained by extrapolating the brown colour baseline using an exponential fitting to obtain the estimated baseline value at 500 nm. Although the correlation between these two methods was good (R^2^ = 0.8914, *p* < 0.001), the regression equation showed a slope significantly different from 1 (95% confidence interval between 0.3290 to 0.5137), showing that these two methods of calculating the pinking susceptibility are not equivalent in the resulting value for pinking susceptibility. It is common practice to use the cut-off value of 5 for both methods, and therefore the use of this cut-off value for deciding the pinking susceptibility of the wines will yield different conclusions depending on the method employed. For example, a cut-off value of 5 in the first method corresponds to a measured value of 7.0 in the second method; on the other hand, a cut-off value of 5 in the second method corresponds to a measured value of 0.30 in the first method, and therefore this means that the wine would not be susceptible for pinking when using the first method (Figure 3a). The amplitude of the first derivative spectra was evaluated as an alternative procedure for removing the baseline due to the browning of the wine solution during the oxidation treatment and the results obtained were compared to the two classic methods for measuring the pinking susceptibility by measuring the increase in absorbance at 500 nm before and after oxidation and the values (Figure 3b) and those obtained by extrapolating the brown colour baseline using an exponential fitting to obtain the estimated baseline value at 500 nm (Figure 3c). As can be observed the correlation between the first derivative method was higher with that of the background correction method (Figure 3c) when compared to the difference in absorbance before and after oxidation (Figure 3b). This is expected as the first derivative of the UV-Vis spectra is known to remove the spectra background, the same purpose of the background corrected method of Simpson [8]. Using Simpson’s cut-off value of 5, the cut-off value for the first derivative spectroscopy method would be −0.00010.

The variation in the a* value and the a* value after the forced oxidation treatment of white wines was compared to the pinking susceptibility values obtained from the two classic methods proposed by Simpson [8] and with the use of the first derivative spectra (Figure 3d, 3e, and 3f, respectively). The classic method of Simpson [8] showed a moderate correlation with the variation of the a* value, with the subtraction of the absorbance value at 500 nm before and after oxidation explaining only 71.5% of the variation observed in the a* values before and after oxidation treatment (Figure 3d). On the other hand, the classic method of Simpson [8] using the extrapolated baseline value at 500 nm and the amplitude of the first derivative spectroscopy explained 97 and 93% of the variation in the a* values of the wines after oxidation treatment, respectively (Figure 3e and 3f). Therefore, both these methods can capture the variation in the a* value of wines after oxidation. In fact, most probably the a* value should be used for evaluating the pinking susceptibility of wines. In this case, and extrapolating the cut-off value of 5 from Simpson, the corresponding cut-off value using the a* values corresponds to 0.6.

### 3.2. Absorption Species in Wines after Forced Oxidation: Second Derivative Spectra

**Figure 4:**
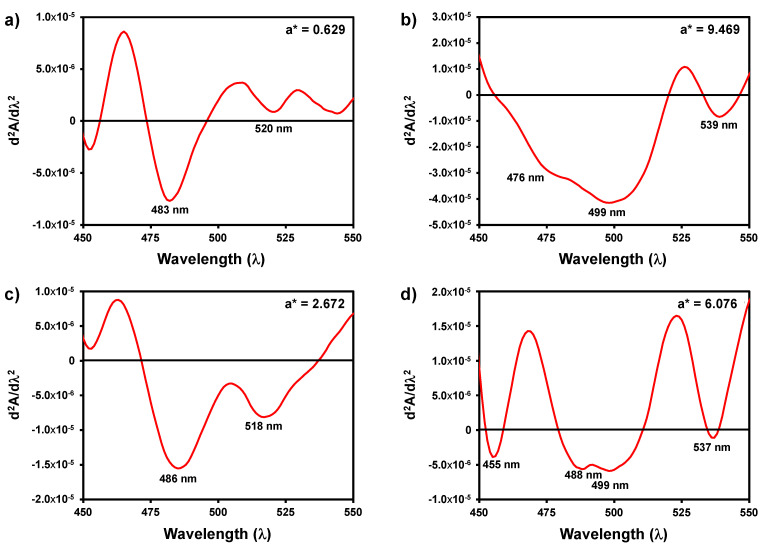
Second derivative visible spectra of four wines after induced oxidation with hydrogen peroxide at room temperature during 24 h. (**a**) Wine sample 2; (**b**) wine sample 4; (**c**) wine sample 7; (**d**) wine sample 11.

In order to access the diversity of chromophores in the 450–550 nm range that are responsible for the appearance of the pink colouration after forced oxidation, the second derivative spectra of different wines obtained after forced oxidation were calculated. As can be observed, the second derivative spectra of the wines forced to oxidise yield in most of the cases more than one band in with different wavelength maxima and with different relative intensities (Figure 4). These results suggest the possible formation of different chromophores in the different wines after forced oxidation.

### 3.3. Perception Threshold for Pink Colouration

To evaluate the correlation between the visual perception of pinking and the colour defined by the CIEL*a*b* parameters, five wines characterised by a different intensity of the yellow colour (b*) were analysed after progressive additions of a deep coloured red wine. For each series, a visual evaluation was performed by nine judges to identify when the pink colour could be detected. Panel consensus on the wine pink colour detection was accessed through the percentage of variance explained by the first principal component (PC) obtained by principal component analysis (PCA) [25]. The variance explained by the first PC was 77%, yielding the C-indexes of 3.4. For a sensory attribute, the higher the explained variance and C-index, the closer this attribute is to unidimensionality, indicating the consensus of the panel in the perception of this attribute. Similar values have been reported for trained panels assessing different attributes and different products [25].

**Figure 5:**
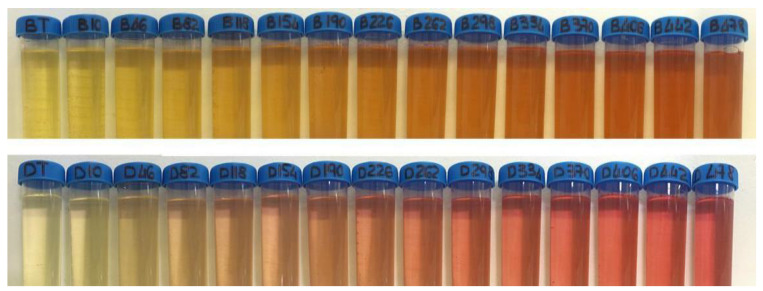
Representative image of two series of white wines with an increasing volume of a deep red wine added. All additions (10 to 478 µL) were made on a final volume of 50 mL.

In the two representative series shown in Figure 5, it is evident how the yellow starting component of the wine greatly influences the perception of the pink colour. With the same amount of deep red wine added, white wines characterised by a lower yellow component (b*) are those that appear more pink, while a more brownish colour is evident in wines characterised by a higher initial yellow (b*) component.

**Figure 6:**
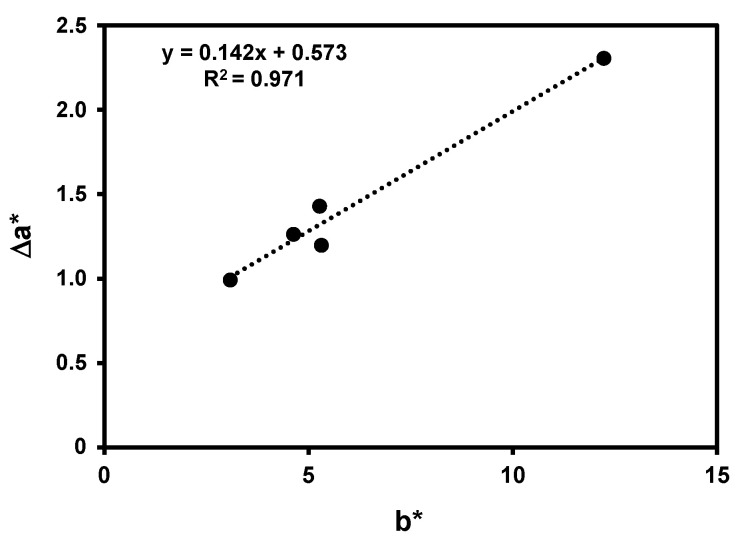
Variation of the a* value needed for the visualisation of the pink colouration in white wines with added red wine for white wines with different b* values.

A significant correlation was observed between the Δa* values needed for the pinking colouration observation by the judges and the b* values of the wines (Figure 6). Therefore, these results show that the cut-off value that should be used for pinking susceptibility is dependent on the yellow component of the white wine (b*). For a light-yellow white wine (b* value of 3.080), the value of the increase in the a* value was 0.992; nevertheless, for a high coloured white wine (b* value of 12.232), the increase in the a* value was 2.3059. For this last wine, some panelists (four in nine) identified the colour change not as pinking but as brownish wine.

### 3.4. Evaluation of a Rapid Method for Measuring the Pinking Susceptibility of White Wines

**Figure 7:**
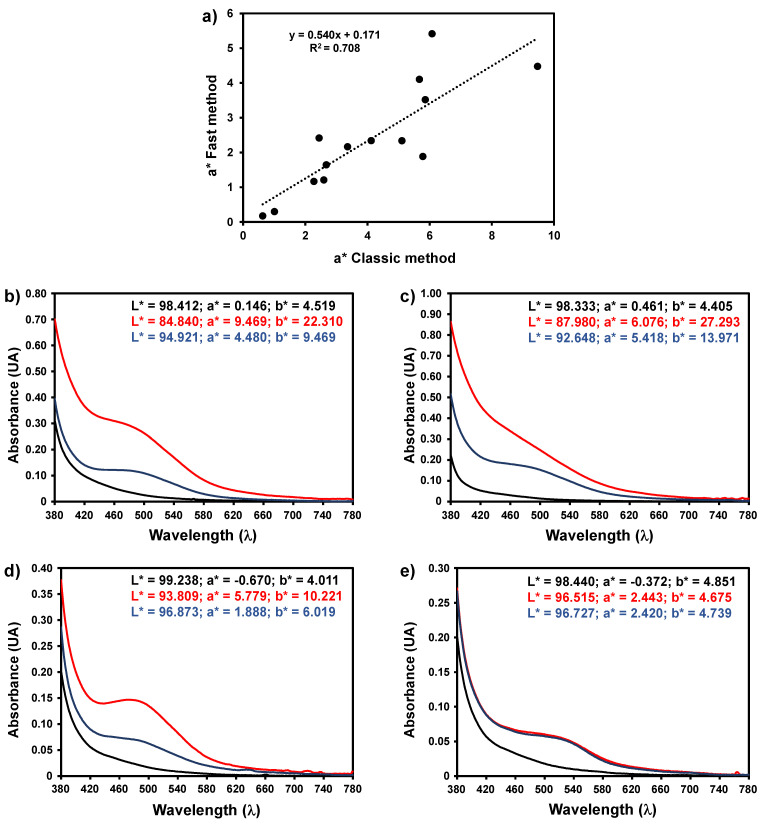
(**a**) Correlation between the classic and fast method for determination of the pinking susceptibility of white wines using the a* value; (**b**) visible spectra of wine sample 4 before (black line) and after oxidation using the classic method (red line) and fast method (blue line); (**c**) visible spectra of wine sample 11 before (black line) and after oxidation using the classic method (red line) and fast method (blue line); (**d**) visible spectra of wine sample 13 before (black line) and after oxidation using the classic method (red line) and fast method (blue line); (**e**) visible spectra of wine sample 14 before (black line) and after oxidation using the classic method (red line) and fast method (blue line).

The classic method of inducing oxidation of white wines for measuring the pinking susceptibility varies according to the country concerning the volume of wine used and the hydrogen peroxide amount added for oxidation [27]. Nevertheless, all the methods are time consuming, normally taking 24 h to complete. In some laboratories and cellars, a rapid method is implemented where it decreased the analysis time (30 min) by increasing the reaction temperature during oxidation to 60 °C. As can be observed in Figure 7, a moderate to good correlation was observed between the two methods; nevertheless, the rapid method yielded a* values lower than the classic method, about half the value. As observed for the classic method there was a very good correlation between the a* values and the value obtained by measuring the absorbance at 520 nm after baseline correction (R^2^ = 0.967) and the amplitude of the first derivative spectra (R^2^ = 0.934) for the fast method. A lower, but also good correlation was observed between the Δa* value and the measurement of the absorbance at 520 nm before and after the induced oxidation (R^2^ = 0.853) for the fast method.

**Figure 8:**
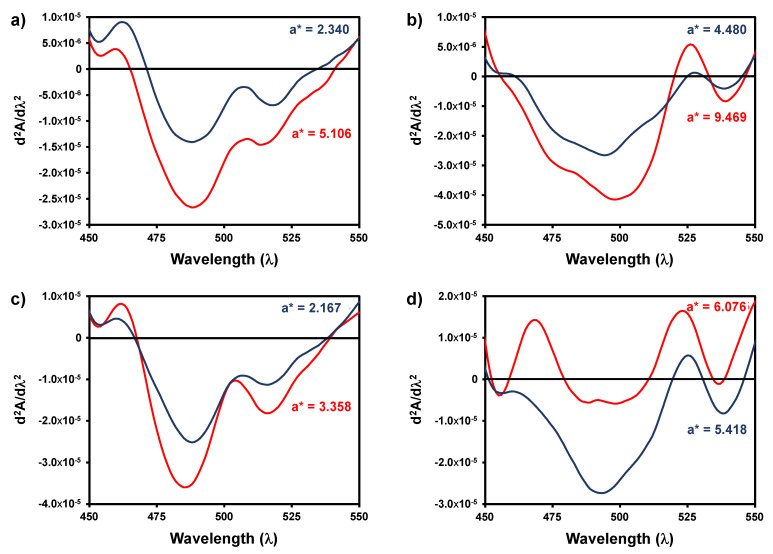
Second derivative visible spectra of four wines after induced oxidation with hydrogen peroxide at room temperature during 24 h (red line) and after oxidation at 60 °C during 30 min (blue line). (**a**) Wine sample 3; (**b**) wine sample 4; (**c**) wine sample 8; (**d**) wine sample 11.

In order to determine if the differences in the a* values could be due to the formation of different chromophores during the high temperature and low time of heating, the second derivative spectra for the classic and rapid method were compared (Figure 8). As can be observed the profile of the second derivative spectra of the visible spectra for the two methods were similar, although with a lower intensity. Therefore, the same chromophores are being formed, nevertheless, with a lower intensity.

## 4. Discussion

The change in the colour of young white wines produced under reductive conditions due to the appearance of a pink colour when wines are exposed to oxidative conditions is one of the most problematic white wine defects at present. When white wines susceptible to pinking are bottled, the appearance of this discolouration will reduce the perceived wine quality. Therefore, before bottling white wines are normally tested for pinking susceptibility by forced oxidation using hydrogen peroxide, and the appearance of the pink colour is measured by measuring the absorbance at 500 nm. Nevertheless, as reported by Simpson [8], these conditions also determine an increase in the signal at 420 nm commonly used to evaluate another chromatic phenomenon typical of white wines: browning. The browning alteration normally results is a broad band extending through the visible region and therefore may interfere with the measurement of the pink colour at 500 nm. Therefore, a method proposed by Simpson [8] to avoid this interference consists of extrapolating the baseline caused by browning after forced oxidation. The cut-off normally used for predicting the appearance of visible pinking colouration after bottling cannot be the same when using the simple subtraction of the absorbance at 500 nm before and after forced oxidation and by subtracting the extrapolated baseline at 500 nm after forced oxidation as there are significant differences in the absolute values obtained by the two methods. As an alternative method for removing the background due to the browning process, the amplitude of the first derivative spectra in the 450–550 nm range can be used and has a good correlation with the values obtained by subtracting the extrapolate background at 500 nm (R^2^ = 0.927); therefore, first derivative spectroscopy seems to be a more straightforward approach for eliminating the background caused by browning due to the forced oxidation conditions. When these three methods are compared with the CIEL*a*b* results, namely with the a* value that measures the red component of the tested wines after forced oxidation, it can be concluded that the variation in a* value only accounts for 71% of the variation in the absorbance at 500 nm. On the other hand, for the extrapolated background method, the a* value of the forced oxidised wines accounts for 97% of the variation measured by the method proposed by Simpson [8]. On the other hand, a linear relationship was also observed with the a* value and the amplitude of the first derivative spectra in the 500 nm region (R^2^ = 0.968). As the CIEL*a*b* allows the reproduction of the visual sensation of colour perceived by observers through the use of the three chromaticity coordinates: lightness (L*), red/green colour component (a*), and blue/yellow colour component (b*), and offer a more precise description of the wine colour [19,20], the CIEL*a*b* values should be a better description of the colour of the wines after oxidation.

The chromophores formed during the forced white wine oxidation with hydrogen peroxide in different wines analysed were different as observed in the second derivative spectra of the wines, raising the hypothesis that in different wines the compounds responsible for pinking can be different, or the precursors are the same but the wine composition can affect the final product formed after oxidation. In this sense, it is necessary to determine the cut-off value in this system to determine the probability of the pinking being observable by the human eye. The pink colouration visualisation given by the a* value depends on the b* of the wines, with the change in the a* value needed for the visualisation of red colour in the wines being linearly related with the b* value. Therefore, these results show that no universal cut-off value for predicting the pink visualisation should be used.

Using a higher temperature, it is possible to induce the pink colour formation in white wines in a much shorter time (30 min). Although a moderate to good correlation is observed between the a* values between the two methods, the intensity of colour formation in the rapid method is lower than that observed for the classic method. This could be due to the incomplete conversion of the compounds responsible for the pinking in the much lower time of reaction or due to a lower formation of interfering compounds as the browning of these wines was also lower when compared to the classic method of oxidation proposed by Simpson [8].

## 5. Conclusions

The CIEL*a*b* method using the a* value after oxidation seems to be the most appropriate method to measure the pinking susceptibility of white wines. The cut-off value to be used is dependent on the b* values of the wines under study as the yellow colour of the wines affect the perception of the red colour. The cut-off value of 5 for the method proposed by Simpson [8] is one of the widely used value in the wine industry for accessing the pinking susceptibility of white wines with decades of use. Therefore, this value was used to calculate the cut-off values for the other methods yielding a cut-off value of a* > 0.6 when using the CIEL*a*b* method. The method for measuring the pinking susceptibility using the a* value is correlated with the values obtained for pinking susceptibility using the amplitude of the first derivative spectra and the method using the extrapolated baseline after forced oxidation. For the amplitude of the first derivative spectra, values higher than −0.00010 can be used as a cut-off value for accessing the pinking susceptibility of white wines. Nevertheless, as the visualisation of the pink colour in wines with different b* values is dependent on the b* value, a unique cut-off value for all wines is not advised. For each type of wine, the cut-off value should be adjusted by wine producers taking into account the specific wine characteristics, namely, their b* values. Different chromophores are formed in different wines under forced oxidation conditions; nevertheless, this does not necessarily mean that different compounds responsible for the pinking phenomena are originally present in different wines, as the compounds formed after forced oxidation conditions might be also dependent on the composition of the wine matrix such as its phenolic composition, among others. The fast method for developing pinking, although presenting a good correlation with the classic method proposed by Simpson [8], yielded lower values, probably related to the incomplete conversion of the pinking chromophores under the reaction conditions used.

## Figures and Tables

**Table 1:** Wine samples used in this study.

	Wines	Origin	Vinification
1	Prosecco base	Northern Italy	Classic
2	Prosecco base	Northern Italy	Classic
3	Generic	Southern Italy	Classic
4	Sauvignon Blanc	Moldova	Reductive
5	Blend (Catarratto + Sauvignon Blanc + Vermentino)	Southern Italy	Classic
6	Blend (Catarratto + Pinot Grey + Muscat)	Southern Italy	Classic
7	Blend (Chardonnay + Pinot Grey + Vermentino + Grillo)	Southern Italy	Classic
8	Blend (Grillo + Sauvignon Blanc + Vermentino + Zibibbo)	Southern Italy	Classic
9	Sauvignon Blanc	Moldova	Reductive
10	Sauvignon Blanc	Moldova	Reductive
11	Pinot Grey	Moldova	Reductive
12	Sauvignon Blanc	Northern Italy	Reductive
13	Chardonnay	Northern Italy	Reductive
14	Generic	Northern Italy	Reductive

## Data Availability

Not applicable.

## Supplementary Materials

The following are available online at https://www.mdpi.com/2304-8158/10/3/553/s1, Table S1. Conventional oenological parameters of the wines used in this study.
