# Supplementary material for: Alternative Methods for Measuring the Susceptibility of White Wines to Pinking Alteration: Derivative Spectroscopy and CIEL*a*b* Colour Analysis
Source: Foods. 2021 Mar 7;10(3):553. doi: 10.3390/foods10030553 (PMC8001532; doi:10.3390/foods10030553)
Supplement: Supplementary file 1 [file foods-10-00553-s001.pdf]

Supplementary Materials

Alternative methods for measuring the susceptibility of white wines to pinking alteration: Derivative spectroscopy and CIEL*a*b* colour analysis

Fabrizio Minute [1]. Federico Giotto[1]. Luís Filipe-Ribeiro[2]. Fernanda Cosme[2]and Fernando M. Nunes [2*]

[1] Giottoconsulting srl. 31051 Follina. Italy; fabrizio@giottoconsulting.it; federico@giottoconsulting.it
[2] CQ-VR – Chemistry Research Center – Vila Real. Food and Wine Laboratory. Department. University of Trás-os-Montes and Alto Douro. 5000-801 Vila Real. Portugal ; fmota@utad.pt; fcosme@utad.pt; fnunes@utad.pt
* Correspondence: fnunes@utad.pt (F.M.N.)

Table 1. Conventional enological parameters of the wines used in this study.

	Wine sample nº													
	1	2	3	4	5	6	7	8	9	10	11	12	13	14
Specific gravity 20°C/20°C	0.994	0.994	0.991	0.991	0.992	0.992	0.992	0.992	0.992	0.991	0.99	0.991	0.991	0.992
Alcohol strength by volume (% v/v a 20°C)	10.04	9.93	13.16	13.89	12.58	12.45	12.71	12.88	12.41	13.75	14.07	12.94	13.67	13.22
Glucose + Fructose (g/L)	0.62	0.54	0.76	0.65	0.54	0.52	0.51	0.55	< 0.10	0.97	0.7	0.8	0.77	3.06
Reducing substances (g/L)	3.5	3.5	4.1	3.7	3.8	3.8	3.8	4	3.3	4.1	3.9	3.7	3.8	5.6
Non reductive dry matter (g/L)	19	18.4	19.8	21.4	21.1	20.4	20.1	20.4	21.5	20.2	20.9	18.5	20.7	19.7
Total dry matter (g/L)	19.6	19	20.6	22	21.6	20.9	20.6	20.9	21.6	21.2	21.6	19.3	21.4	22.8
Total acidity (tartaric acid) (g/L)	4.19	3.93	6.07	6.01	6.37	5.44	5.45	5.75	6.08	6.24	5.74	6.46	6	5.82
pH	3.47	3.55	3.15	3.34	3.18	3.33	3.26	3.21	3.27	3.22	3.33	3.16	3.3	3.28
Volatile acidity (g/L)	< 0.10	< 0.10	0.1	0.38	0.17	0.18	0.11	0.18	0.34	0.26	0.27	0.31	0.38	0.25
Malic acid (g/L)	0.64	0.3	1.27	1.51	1.63	1.64	1.19	1.39	1.36	1.54	1.54	2.03	2.49	2.12
Lactic acid (g/L)	1.12	1.37	< 0.20	0.22	< 0.20	< 0.20	< 0.20	< 0.20	< 0.20	< 0.20	< 0.20	< 0.20	< 0.20	< 0.20
Tartaric acid (g/L)	2.7	2.71	3.56	3.45	3.31	2.68	3.04	3.17	4.09	3.61	3.38	3.27	2.89	2.67
Glycerol (g/L)	4.93	4.69	5.72	6.37	6.05	5.6	5.21	5.86	5.6	6.17	6	6.33	5.69	6.38
Potassium (g/L)	1.11	1.21	0.78	0.96	0.87	0.98	0.86	0.82	0.95	0.92	0.91	0.81	0.93	0.87
Free sulfur dioxide (mg/L)	5	2	6	8	9	10	23	23	2	22	11	18	17	10
Total sulfur dioxide (mg/L)	54	51	34	65	56	67	67	76	70	99	52	101	116	56
